# Prevalence of telogen effluvium hair loss in COVID-19 patients and its relationship with disease severity

**DOI:** 10.25122/jml-2021-0380

**Published:** 2022-05

**Authors:** Shahram Seyfi, Robabeh Alijanpour, Zeinab Aryanian, Khadijeh Ezoji, Mahdi Mahmoudi

**Affiliations:** 1. Department of Anesthesiology, Babol University of Medical Sciences, Babol, Iran; 2. Iranian Medical Laser Association, Babol, Iran; 3. Autoimmune Bullous Diseases Research Center, Tehran University of Medical Sciences, Tehran, Iran; 4. Social Determinants of Health Research Center, Health Research Institute, Babol University of Medical Sciences, Babol, Iran; 5. Clinical Research Development, Unit of Ayatollah Rohani Hospital, Babol University of Medical Sciences, Babol, Iran

**Keywords:** Telogen, hair loss, COVID-19

## Abstract

COVID-19 is a concerning global pandemic. Common manifestations are fever and respiratory symptoms. In addition, recent studies reported dermatological manifestations as extrapulmonary signs. One of these is telogen effluvium which is related to post COVID-19 comorbidities. The aim of this study was to assess the prevalence of telogen effluvium among COVID-19 patients. This observational cross-sectional study included 198 patients who were admitted for COVID-19. The PCR test was performed to detect positive cases. After discharge, all patients were interviewed about hair loss. Of these patients, 79 were male (39.9%), and 119 were female (60.1%). The age ranged from 18 to 85 years old. 48 patients showed hair loss. Telogen effluvium (TE) is one of the consequences of the COVID-19 pandemic. COVID-19 leads to more medications and stress situations, which trigger TE.

## INTRODUCTION

COVID-19 pandemic has affected all aspects of medical issues due to its unpredictable characteristics. All concerning issues are related to its critical pulmonary and cardiovascular symptoms. However, the cutaneous manifestation of COVID-19 could be the initial presenting sign of COVID-19, as reported in several articles [1]. The COVID-19 emergency and restrictions had negative psychological effects, including anxiety and stress. The psychological reactions facilitate the neurotransmitters, neuropeptides, and hormones released, which promote changes in the hair cycle development from anagen to telogen phase [2]. In addition, COVID-19 impacts various alopecia-related diseases, including androgenetic alopecia, arieata, and effluvium, because the virus disrupts stress and physiological factors [3]. A study showed that COVID-19 could play a significant role by acting on the transmembrane protease, serine 2 (TMPRSS2) gene, which is an important gene in androgens pathways exacerbating alopecia [4–6].

Telogen effluvium (TE) is one of the most popular alopecia in women, provoked by stressful events, trauma, illness, malnutrition, hormonal imbalance, and drugs. The pattern of hair loss in TE is diffuse, without scars, and involves less than half of the hair. It occurs 2–3 months after the stressful condition, and it is occasionally self-limiting. TE could be chronic if it lasts more than 6 months. The patients who suffer from TE are anxious and usually worry about their hair. Therefore, TE has a dramatic impact on their psychological health and mind [2]. TE results from an abnormal hair cycle in which the growing phase decreases, and follicles enter the telogen phase prematurely. Therefore that leads to increased shedding within months [7]. The psychological impact of hair and skin disorders on patients' quality of life and satisfaction is remarkable, resulting in subsequent anxiety and depression [8, 9].

While common triggering factors for TE are febrile disease, severe infection, and nutritional deficiencies, which are common in COVID-19 patients, it is worth evaluating the prevalence of TE. The study on the cutaneous manifestation of COVID-19 can be useful in determining the severity of the disease. This study aimed to investigate the prevalence of TE in COVID-19 patients.

## MATERIAL AND METHODS

### Research design

This observational cross-sectional study was performed in Rohani hospital of Babol Medical University Of Sciences. We reviewed the medical records of 198 patients admitted due to the COVID-19 positive RT-PCR test.

### Data collection

Some data were collected from their medical records. The demographics data, signs and symptoms of COVID-19, past medical history, drug history, and dermatologic manifestation were reviewed by convenience sampling. Also, disease severity based on radiological and laboratory findings were collected from their medical records. Other information included hair loss, family history of hair loss, daily life habits, theory of bathing, nutrition etc, based on a questionnaire completed by phone after discharge.

### Sample size calculation

Based on the below formula, the sample size required per group was 198. Hence, the total sample size required was 198.


$$n=\frac{Z_{1-\frac{\alpha }{2}}^{2}p(1-p)}{d^{2}}$$

$$\alpha =0.05;{\text{ Z}}_{\text{1-}\frac{\alpha }{2}}=1.96;p=0.005;d=0.004;$$

$$N=\left(1.96\right)2\left(0.005\times 0.995\right)/\left(0.004\right)2=198.$$

### Statistical analysis

Qualitative data were expressed by ratio and percentage, and quantitative data by mean and standard deviation. Data were analyzed using SPSS software version 22 using T-test and Chi-square. Significant levels were considered at P≤0.05.

## RESULTS

198 patients were included in this cross-sectional study. 138 confirmed COVID-19 patients underwent TE diagnosing whose data were extracted from the hospital record concerning age and gender. Of these patients, 79 were male (39.9%), and 119 were female (60.1%). The age ranged from 18 to 85 years old. The demographic data are summarized in Table 1. No correlation was seen between hair loss and age or sex. There was a significant correlation between positive COVID-19 test and hair loss among patients. 42 patients received Favipriavir and 41 Remdesevier.

**Table 1:** Clinical investigations of research participants.

Parameters			Total (n=198)	P-value*
**Sex**		**Male**	79 (39.9%)	0.531
		**Female**	119 (60.1%)	0.519
**Age**			56.04±15.25	0.801
**Favipiravir**			42 (21.2%)	0.843
**Remdesevier**			42 (21.2%)	0.072
**Corona positive PCR**			138 (69.7%)	≤0.01
**Hair loss**	**No**		150 (75.8%)	0.0512
	**Yes**		48 (24.2%)	0.145

* – p˂0.05.

## DISCUSSION

The common symptoms of COVID-19 are fever and pulmonary manifestations. In addition, telogen effluvium was reported in some studies associated with COVID-19 [10–12]. Telogen effluvium is a kind of diffuse hair loss several weeks after a stressor. The provoking factors include systemic infection, pregnancy, psychological trauma, surgery, malnutrition, and drugs which result in premature termination of the anagen phase and hair shedding. TE is a self-limited disease, and scarring is rare [7].

Olds et al. study reported 10 cases of TE post-COVID-19 infection. The majority were female, which is not consistent with this study. Most patients had a severe disease that required hospitalization and systemic medications, which is consistent with our patients. One of the strengths of this study is the sample size. Based on our knowledge, previous studies were case reports or case series [10, 11].

Telogen effluvium is the most common diffuse hair shedding because of the premature termination of anagen and hair follicle entry into catagen. The telogen effluvium was one of the catastrophic impacts of the influenza pandemic in 1918. The mean duration of hair loss was 9 weeks. It seems that this period is shorter in COVID-19 patients. COVID-19 could be considered a main trigger of severe hair shedding. SARS-CoV-2 patients revealed higher levels of proinflammatory cytokines (tumor necrosis factor, interleukin 1b, interleukin 6, and interferon types 1 and 2), which may explain skin manifestations associated with infection, such as livedoid vasculopathy, urticaria, COVID toes, and a chicken pox-like rash. As matrix cells are destroyed during an immune response, an overabundance of interferons can lead to TE formation. This is why cytokine storms need to be avoided wherever possible. Other factors, including lack of scalp hygiene in hospitals and medications, should be assessed [13].

The cytokines had higher levels in severe COVID-19, according to previous studies. The higher levels of cytokines may have a relationship with a higher risk of TE. The inflammatory cytokines, including IL-6, TNFα, IL-1β, and IFNg, develop the catagen cycle in experimental studies [14]. Furthermore, anticoagulant proteins are decreased due to the anticoagulation cascade in response to COVID-19. It may cause microthrombi formation and obstruct hair follicle blood supply. These factors could be considered precipitating factors of TE after COVID-19 infection [15, 16].

The symptom of hair loss developed several weeks after the clinical manifestation of COVID-19. Di Landro et al. study reported that all patients had a normal range of iron, ferritin, vitamin B12, and thyroid function tests. However, their principal complaint was hair thinning and hair loss [13]. Therefore, it supports the hypothesis of post-COVID-19 complication, which is related to dermatological manifestation.

As mentioned, TE was seen in both sexes in our studies, while in the Mieckowska et al. study, all patients were female with no history of hair loss. The median age was 55, and they experienced excessive hair loss several weeks to months after infections. Telemedicine was the common way to diagnose hair loss in their study and this study [17, 18]. Shome et al. reported that 20 adult patients (all women) were included in the study, starting a few weeks after recovery from COVID-19 infection and continuously presenting with TE for more than 6 months. A solution of 1.5 ml of the hair growth factor formulation QR678 Neo^®^ was administered to the scalp per session. A total of 8 sessions were performed (one session every 4 weeks). Outcomes were assessed at baseline, 1 month after the 4^th^ semester, and 1 month after the 8^th^ semester. Most of the patients showed a significant reduction in hair loss; 89% of patients showed excellent hair growth. The global score of the photographic evaluation showed a significant improvement, which persisted even after treatment. The video microscopic evaluation showed an increase in the number of hairs after the 8^th^ session (mean=29.32), which improved further after treatment. Subjective evaluation scores for overall hair growth, hair appearance, reduction in scalp visibility, and hair loss were 4, 4.5, 4.25, and 5, respectively [19]. Sharquie et al. showed that 29 patients aged 22 to 67 years with an average and SD of 41.3×11.6 years with 36 (92.3%) females and 3 (7.69%) males. All patients diagnosed with TCA were enrolled in this study and had a previously laboratory-confirmed diagnosis of SARS-CoV-2 infection. 15 (38.46%) patients reported mild symptoms, 24 (61.53%) patients had moderate illnesses, and no patient required hospitalization. They all suffered from excessive hair loss within 2–3 months of infection. Tests were strongly positive (>10–50% on average, 35% of hair was pulled from the scalp) [20]. Hossuni et al. performed a systematic review involving 465 patients diagnosed with acute TE. The mean age was 44 years, and 67.5% were women. The most common trichoscopic findings are a decrease in hair density, empty follicles, or the regeneration of short hairs. The average duration from the onset of COVID-19 symptoms to the appearance of acute TE is 74 days, earlier than classic acute TE. While most patients recover from hair loss, some have persistent hair loss. In the context of the COVID-19 epidemic, our results highlight the need to consider the possibility of COVID-19 acute TE in hair loss patients with a history of COVID-19 infection. Despite the self-limiting condition, hair loss is a stressful expression of COVID-19. Identifying COVID-19 infection as a possible cause of acute TE can help doctors advise patients, thereby relieving them of unwanted stress [21]. Strace et al. provided data on 128 patients. Telogen alopecia was seen in 66.3% of patients, trichodynia – in 58.4%. Trichodynia was associated with telogen effluvium in 42.4% of cases and anosmia and eosinophilia in 66.1% and 44.1% of cases, respectively. The majority of patients (62.5%) developed hair-related signs and symptoms within the first month of being diagnosed with COVID-19, and 47.8% of patients developed hair-related signs and symptoms after 12 weeks or more [22].

There are several limitations to this study. First of all, the sample size was limited and the study is only retrospective. Based on our knowledge, this is the first cross-sectional study investigating TE triggered by COVID-19 in Iran. Compared with other studies, we had less demographic data. TE can be caused due to medications that cannot be ruled out. TE can occur as a result of hydroxychloroquine, azithromycin, or other drugs used in COVID-19 treatment. The mental and psychological effects of the pandemic are another critical source of stress; therefore, TE may be increased overall [17].

## CONCLUSION

Telogen effluvium is one of the consequences of the COVID-19 pandemic. COVID-19 leads to more medications and stress situations, which trigger TE. Physicians should be aware of this delayed TE. To the best of our knowledge, this is the first cross-sectional study with 48 positive cases. In our study, none of the patients had previous TE. Further studies for investigating and clarifying the pathogenesis are required. It can also be suggested that the stress caused by COVID-19 disease could be a cause of hair loss.
